# Matching treatment options for risk sub-groups in musculoskeletal pain: a consensus groups study

**DOI:** 10.1186/s12891-019-2587-z

**Published:** 2019-06-01

**Authors:** Joanne Protheroe, Benjamin Saunders, Bernadette Bartlam, Kate M. Dunn, Vince Cooper, Paul Campbell, Jonathan C. Hill, Stephanie Tooth, Christian D. Mallen, Elaine M. Hay, Nadine E. Foster

**Affiliations:** 1 Primary Care Centre Versus Arthritis, Research Institute for Primary Care & Health Sciences, Keele University, Staffordshire, ST5 5BG UK; 2 Lee Kong Chian School of Medicine, Nanyang Technological University, 50 Nanyang Ave, Singapore, 63979 Singapore; 3 Keele Clinical Trials Unit (CTU), David Weatherall Building, Keele University, Staffordshire, ST5 5BG UK

**Keywords:** Musculoskeletal pain, Primary care, Consensus group methods, Nominal group technique

## Abstract

**Background:**

Musculoskeletal (MSK) pain represents a considerable worldwide healthcare burden. This study aimed to gain consensus from practitioners who work with MSK pain patients, on the most appropriate primary care treatment options for subgroups of patients based on prognostic risk of persistent disabling pain. Agreement was sought on treatment options for the five most common MSK pain presentations: back, neck, knee, shoulder and multisite pain, across three risk subgroups: low, medium and high.

**Methods:**

Three consensus group meetings were conducted with multi-disciplinary groups of practitioners (*n* = 20) using Nominal Group Technique, a systematic approach to building consensus using structured in-person meetings of stakeholders which follows a distinct set of stages.

**Results:**

For all five pain presentations, “education and advice” and “simple oral and topical pain medications” were agreed to be appropriate for all subgroups. For patients at low risk, across all five pain presentations “review by primary care practitioner if not improving after 6 weeks” also reached consensus. Treatment options for those at medium risk differed slightly across pain-presentations, but all included: “consider referral to physiotherapy” and “consider referral to MSK-interface-clinic”. Treatment options for patients at high risk also varied by pain presentation. Some of the same options were included as for patients at medium risk, and additional options included: “opioids”; “consider referral to expert patient programme” (across all pain presentations); and “consider referral for surgical opinion” (back, knee, neck, shoulder). “Consider referral to rheumatology” was agreed for patients at medium and high risk who have multisite pain.

**Conclusion:**

In addressing the current lack of robust evidence on the effectiveness of different treatment options for MSK pain, this study generated consensus from practitioners on the most appropriate primary care treatment options for MSK patients stratified according to prognostic risk. These findings can help inform future clinical decision-making and also influenced the matched treatment options in a trial of stratified primary care for MSK pain patients.

## Background

Musculoskeletal (MSK) pain represents a considerable worldwide healthcare burden [1] and, in the UK, accounts for 14% of all general practice (GP) consultations [2]. Individual GP treatment is highly variable [3], which may be in part due to a lack of confidence in managing these problems [4], as well as MSK pain being given lower priority when compared with other acute illnesses with more medically critical symptoms [5, 6]. A key result of this variability in management is that some patients may, at least initially, receive suboptimal care including inappropriate treatment referrals, whilst others fail to receive appropriate early referral [7, 8].

Current usual primary care for MSK pain in the UK commonly follows a ‘stepped, wait and see’ approach, with patients initially given low intensity and low cost treatments, moving onto higher intensity or more costly treatments if needed [9]. An alternative approach is to stratify patient care according to the patient’s risk of poor outcome, e.g. persistent disabling pain. Stratified primary care has been shown to be clinically and cost effective for low back pain [10–12]. This approach involved the use of a brief self-report tool− the STarT Back tool [13], to identify patients’ risk of persistent disabling pain, and matching risk subgroups (i.e. low, medium and high risk) to appropriate first-line treatment options. Using this approach, patients who need more intensive treatment are identified at the outset, allowing them to be ‘fast-tracked’ to that treatment, whilst patients at low risk can be reassured of their good prognosis and unnecessary treatments can be avoided. The most recent UK National Institute for Health and Care Excellence (NICE) guidelines [14] recommend risk stratification for low back pain in primary care; for instance, using the STarT Back Tool [13].

Given findings that similar prognostic factors predict outcome across different body region pain sites [15], the Stratified Primary Care for Musculoskeletal Pain research programme is developing and testing a new stratified care intervention for patients with the five most common musculoskeletal pain presentations in primary care – back, neck, shoulder, knee and multisite pain. A new prognostic tool developed and validated as part of a linked observation cohort study (*n* = 1890) [16] – the STarT MSK tool™, is used in general practice to stratify patients as low, medium or high in relation to risk of persistent disabling pain, with matched treatment options recommended for each subgroup. The reason for developing a new stratified care model is that whilst the prognostic factors do overlap with STarT Back, it was recognised that the matched treatments needed to reflect the slightly different patient profiles of the STarT MSK Tool’s low, medium and high risk subgroups, as well the more heterogeneous population being targeted, which requires a broader range of recommended matched treatments, for high risk patients in particular. The clinical effectiveness and cost-effectiveness of this approach compared to usual, non-stratified care is currently being tested in a randomised controlled trial (RCT).

This paper reports on an expert consensus groups study, the findings of which informed the matched treatment options tested as part of the trial. The aim was to gain consensus from a range of practitioners who work with MSK pain patients on the most appropriate primary care treatment options, in a UK National Health Service (NHS) context, for patients consulting with the five pain presentations of interest across the three different risk subgroups, based on prognostic risk of persistent disabling pain. Consensus group methods are particularly useful in fields where scientific evidence is uncertain or inconclusive [17]. The approach was therefore well-suited to the aims of this study, as a systematic review conducted earlier in the research programme revealed a lack of robust evidence on the effectiveness of different MSK treatment options [18]. Whilst the study has a UK focus, the role of GPs and primary care physiotherapists in providing first line management for MSK conditions is common to many countries; therefore, findings can have wider applicability for informing practice in other non-UK settings.

## Methods

### Study design

This study uses a consensus groups method; an approach commonly employed in the development of healthcare interventions, due principally to their capacity to ‘harness the insights of appropriate experts to enable decisions to be made’ [17]. The consensus group method used in this study is Nominal Group Technique (NGT). Originally developed by Delbecq, van de Ven and Gustafson [19], NGT is a systematic approach to building consensus using structured in-person meetings of experts. NGT follows a distinct set of stages that involves participants initially generating ideas on a particular issue or problem, which are then individually rated by participants. Following this, the results of these ratings are discussed within the group, and then re-rated individually by participants, who are given the opportunity to change their scores in light of group discussions. This process can be repeated several times until consensus is achieved. The threshold used to signal an acceptable level of consensus varies across NGT studies depending on the aims and design adopted; the threshold we adopted is discussed later; see ‘Assessing consensus’. NGT can be adapted, for instance through use of a ‘pre-elicitation technique’ [20], that is, providing participants with a summary of existing evidence to inform their decision-making. The pre-elicitation technique was used in this study due to the wide range of literature on MSK treatments (see below for further explanation). The stages of the NGT process are described in detail below.

### Participants

We invited stakeholders from a range of clinical backgrounds, including general practice, physiotherapy, rheumatology, orthopaedics, clinical psychology and pain medicine (see Table 1 for participant characteristics). Participants were identified through existing clinical and research networks in the UK, and were invited via email. Dates for the meetings were arranged at the convenience of the practitioners. The same practitioners were invited to all three consensus group meetings (see Results below).

**Table 1:** Participant Characteristics: Breakdown of clinical expert participants by gender and professional background across the three consensus group meetings

	Consensus group meeting 1 (n=18)	Consensus group meeting 2 (n=16)	Consensus group meeting 3 (n=12)
Female/ Male	8/10	7/9	5/7
General Practitioners	4	4	4
Physiotherapists specialising in MSK pain	5	5	3
Rheumatologists	3	1	1
Clinical Psychologist	1	1	1
AHP (Allied Health Professionals) clinical coordinator	1	1	1
Therapy pathway manager	1	1	1
Pain medicine consultant	1	1	0
Clinical Research Consultant	1	1	1
Spinal Surgeon	1	1	0

### Ethics

Ethical approval for the study was granted by the Keele University Ethical Review Panel (ref: ERP319). Participation in the study was entirely voluntary. Participants’ travel expenses were reimbursed, but beyond this they received no financial incentive for taking part. Written consent was obtained prior to each NGT meeting.

### Nominal group meetings

Three NGT meetings, each focusing on one of the three patient risk subgroups (low, medium, high risk of persistent disabling pain), were conducted between April–May 2015. Groups were convened at Keele University. Meeting 1 focused on agreeing matched treatment options for patients at low risk of persistent disabling pain, for all five MSK pain presentations. Meetings 2 and 3 focused on matched treatment options for patients at high and medium risk, respectively.

**Fig. 1:**
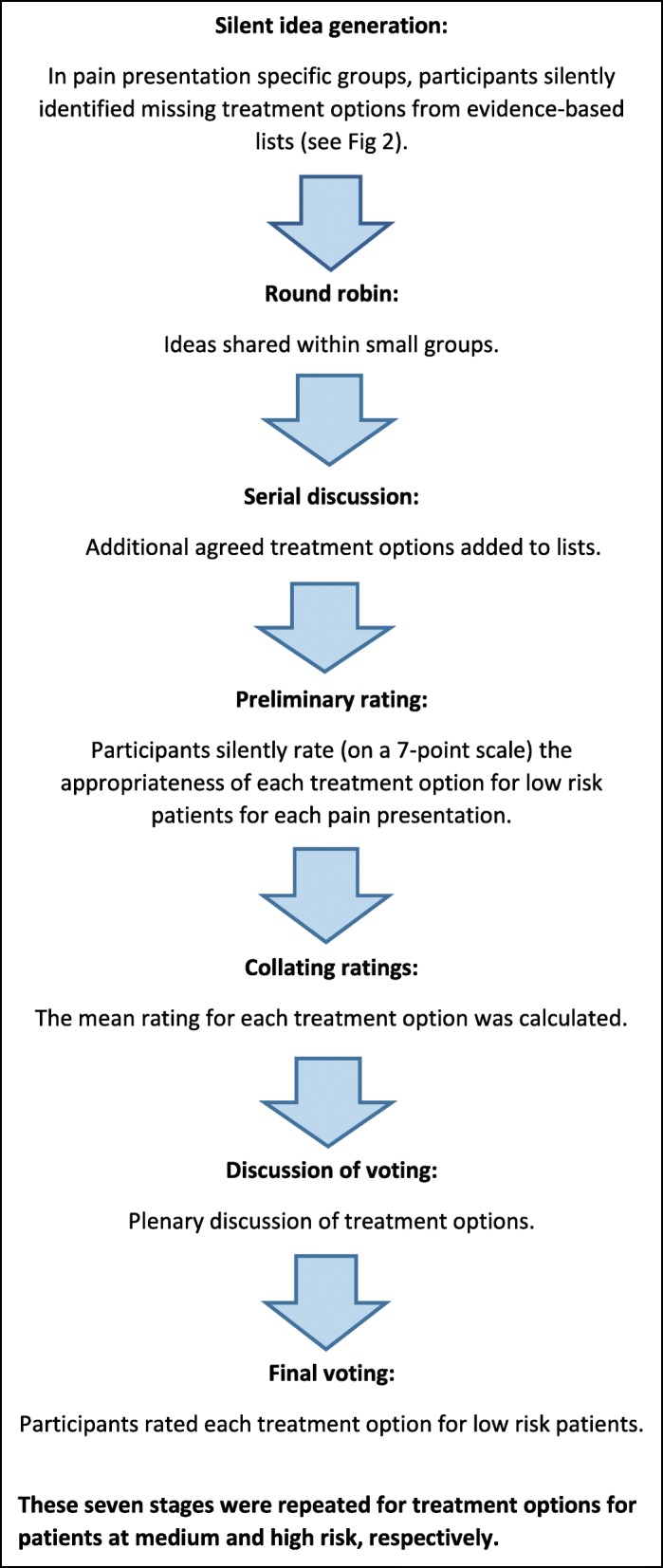
Stages of Nominal Group Technique (NGT): Outline of the stages followed in each of the three consensus group meetings

**Fig. 2:**
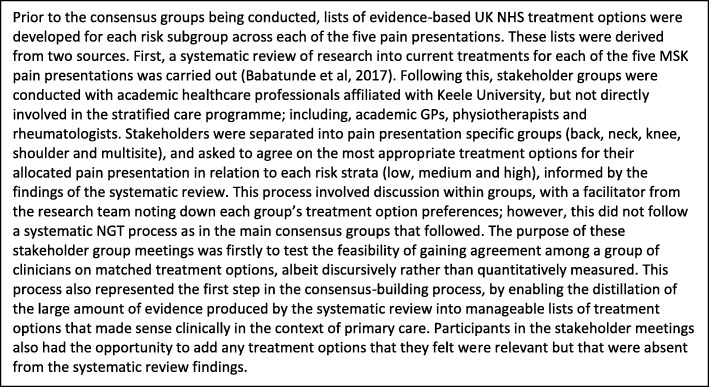
Deriving a priori treatment option lists: Description of the process for developing lists of UK NHS evidence based treatment options for each risk subgroup across each of the five pain presentations prior to the consensus group meetings. These lists were used as pre-elicitation information to inform the practitioners’ decisions

The meetings each followed the same set of stages (see Fig. 1). First a facilitator provided a brief study overview and outlined the aims and stages of the consensus process. Facilitators were members of the research team and did not participate in the group discussions. The same facilitators were used in each meeting. Participants were initially separated into small groups (4–6 participants with a spread of backgrounds and expertise so as to allow for multi-disciplinary discussions) each focusing on one or two MSK pain presentations. Each group was tasked to agree on the most appropriate treatment options for the MSK pain presentation and for the risk subgroup being considered (low, medium or high). Participants were asked to decide on treatment options for a general adult population based on risk of poor outcome, i.e. the patient’s likelihood of experiencing persistent disabling pain. To support this, participants were provided with: a) lists of UK NHS evidence based treatment options, developed for each risk subgroup across each of the five pain presentations (Fig. 2 outlines the development of these lists), and b) patient vignettes exemplifying ‘typical’ patients falling within the relevant risk subgroup, (e.g. for meeting 1, vignettes of patients at low risk). The vignettes were developed using data from patients stratified as low, medium and high risk using the validated STarT MSK tool™ as part of the earlier observational cohort study [16]. Vignettes were used as illustrative examples to give participants information about key characteristics of each patient subgroup in order to inform their decision-making on appropriate treatment options (see Table 2).

**Table 2:** Vignettes of patients at low, medium and high risk: Descriptions of typical patients subgrouped as low, medium and high risk of persistent disabling pain. Vignettes were developed using data collected as part of a linked observational cohort study, and were included to inform the practitioners about key characteristics of each patient subgroup to aid decision making in the consensus group meetings

Low risk patient vignette: back pain	*A 61 year old lecturer visiting her GP with back pain. At the time of completing the questionnaire the pain is 7/10 but usually 4/10 over the last couple of weeks. It is only bothering her slightly and is limiting her activities a little, but she has been taking some over-the-counter painkillers. Rates general health as good. Not feeling anxious or depressed.*
Medium risk patient vignette: multisite pain	*A 45 year-old man consulted his doctor with back, knee and shoulder pain. He rates his current pain as 7/10 and extremely bothersome. He works fulltime as a continuous improvement specialist, and has not had to take any time off work as a result of his pain. He has had his current pain problem for 6-9 months, and is in constant, severe pain. The pain interferes a lot with his enjoyment of life, and his daily activities are limited a little, such as climbing the stairs and carrying groceries. He rates his general health as poor. He feels that there is a very good chance that his pain will be a long-term problem. He has trouble sleeping due to his pain and often feels tired and worn out. He does not feel anxious or depressed when he thinks about his pain, and he feels completely confident that he can cope with his pain in most situations.*
High risk patient vignette: shoulder pain	*A 51 year old full-time service engineer went to his doctor with shoulder pain. He has had his pain for 1-2 years, it is currently 10/10 and extremely bothersome. He is feeling very anxious at the moment, and worries that his pain might become persistent. He is limited a lot in daily activities such as household chores and dressing himself. He feels tired most of the time and is having trouble sleeping. He also has a hiatus hernia.*

Participants first silently reviewed the resources provided in order to identify relevant treatment options they felt were missing from the list of UK NHS evidence based treatment options. Participants were encouraged to focus on first-line actions that could be taken by a GP, rather than treatment decisions made further along the treatment pathway, e.g. specific surgical procedures. Participants took turns to share any additional treatment options within their small group, which were noted on a flipchart by the facilitator. Once all additional ideas had been recorded, each additional treatment option generated through this initial stage (i.e. not including the existing options on the evidence-based lists) was discussed in turn within the small groups. Those that were agreed upon after discussion – in terms of their appropriateness for the given pain presentation and risk subgroup, and those available on the NHS – were added to the treatment options list. Participants then silently and anonymously rated, on a 7-point Likert scale, the appropriateness of each treatment option on the group’s list for the MSK pain presentation and risk subgroup being considered (e.g. appropriateness for patients with shoulder pain classified at low risk). The mean appropriateness score was then calculated for the group for each treatment option on the list by members of the research team. Options were then organised in rank order, i.e. the option with the highest mean ranked number 1, and so on.

All participants then joined together with others for a full group discussion of the treatment options for each MSK pain presentation. The full group was presented with the list of treatment options for each pain presentation in rank order. All participants then had the opportunity to ask questions and suggest further relevant treatments they felt were missing. All suggested options were written on a flipchart and discussed in turn by the full group. Participants then voted via a show of hands as to which treatment options should be added to the final treatment option list (those agreed by more than half of the full group members).

Using the final list, all participants silently and anonymously rated, again on a 7-point Likert scale, the appropriateness of each treatment option (for the MSK pain presentation and risk subgroup). The consensus process described above was repeated for all five MSK pain presentations and for each risk subgroup.

### Assessing consensus

Following the three group meetings, the mean scores were calculated across all participants for each MSK pain presentation and each risk subgroup. A list of treatment options identified for each pain presentation in each risk subgroup was compiled and sent to participants via email to consider and resolve any inconsistencies and approve the finalised list of treatment options. Through discussions within the research team, as well as considering consensus thresholds used in previous NGT studies reported in the research literature, it was decided that a mean rating of > 50% signalled an acceptable level of consensus for a treatment option to be included in the final list. This cut-off point reflected the challenge in gaining consensus from a multi-disciplinary group compared to a group from a single health professional discipline [21–23]. Additionally, we wanted the final treatment option lists to be inclusive so that when these are used by GPs in consultations, the range of available options can allow for patient choice and shared decision-making.

## Results

### Participants

In total, 20 participants attended at least one of the three NGT meetings; however, not all participants were able to attend all three group meetings (group 1: *n* = 18 participants; group 2: *n* = 16; group 3: *n* = 12). Participants had a range of clinical backgrounds, with a mix of experience levels/ length of time in practice (2–27 years in current clinical role; mean = 12 years). They also came from a range of geographical areas within the UK, giving a breath of knowledge on the diversity of existing treatments available within the NHS nationally (see Table 3).

**Table 3:** Geographical spread: Breakdown of clinical expert participants by UK geographical area in which they practise

Geographical area	Number of participants
North West England	4
West Midlands of England	10
South East England	1
North East England	1
London	1
Scotland	2
Wales	1

### Matched treatment options

The following matched treatment options were agreed and are presented in full in Table 4.

**Table 4:** Final agreed matched treatment options: Full list of agreed treatment options for all five pain presentations across the three risk subgroups. Ticked boxes indicate that > 50% agreement was reached for the listed treatment option for the corresponding risk subgroup and pain presentation

L =Low risk; M=Medium risk; H=High risk	Back			Knee			Multisite			Neck			Shoulder		
	L	M	H	L	M	H	L	M	H	L	M	H	L	M	H
Education and advice, including exercise, activity modification, weight loss etc.	✓	✓	✓	✓	✓	✓	✓	✓	✓	✓	✓	✓	✓	✓	✓
Simple oral and topical pain medications limited to those available over the counter	✓	✓	✓	✓	✓	✓	✓	✓	✓	✓	✓	✓	✓	✓	✓
Review by primary care practitioner if not improving after 6 weeks	✓			✓			✓			✓			✓		
Refer to physiotherapy (all modalities)		✓	✓		✓	✓		✓	✓		✓	✓		✓	✓
Refer to MSK interface clinic		✓	✓		✓	✓		✓	✓		✓	✓		✓	✓
Refer to psychosocial intervention or pain management service		✓	✓			✓		✓	✓			✓			✓
Personalised exercise programmes, e.g. personal trainer if available		✓			✓			✓	✓						
Occupational Health/workplace advice		✓	✓		✓	✓					✓	✓		✓	
Address comorbidities, distress and frailty		✓	✓			✓			✓			✓			✓
Atypical analgesia (e.g. amitriptyline, pregabalin, gabapentin)		✓	✓					✓	✓		✓	✓			
Opioids			✓			✓			✓			✓			✓
Refer to supported self-management and locally available community resources e.g. walking group, exercise on prescription							✓								
Refer to expert patient programme			✓			✓			✓			✓			✓
Refer for lifestyle intervention e.g. dietician, slimming world etc.						✓									
Refer for surgical opinion			✓			✓						✓			✓
Corticosteroid injection						✓								✓	✓
Refer to Rheumatology								✓	✓						

#### Treatment options for all risk subgroups

Two treatment options were agreed as appropriate for patients with any of the pain sites and in any risk subgroup: “education and advice” and “simple oral and topical pain medications limited to those available over the counter”. In what follows we present those other options that reached consensus for specific pain-site presentations and/or risk subgroups.

#### Low risk

The following treatment option was agreed for all patients classified as low risk: “review by primary care practitioner if not improving after 6 weeks”. For multisite pain only, a further matched treatment option that reached consensus was: “refer to supported self-management and locally available community resources e.g. walking group, exercise on prescription”.

#### Medium risk

Seven treatment options reached consensus for low back pain, six for multisite pain, and four each for knee pain, neck pain and shoulder pain. For all five pain-site presentations, options included: “consider referral to physiotherapy” and “consider referral to MSK-interface-clinic^1^”. “Occupational Health/work place assessment and advice” was agreed for every pain presentation for medium risk, except for multisite pain. In addition, “consider referral to psychosocial intervention or multidisciplinary pain management service” reached consensus only for back and multisite pain. Three treatment options were agreed in relation to one of the pain presentations only: “address comorbidities, distress and frailty” (back); “refer to rheumatology” (multisite); and “corticosteroid injection” (shoulder). “Personalised exercise programmes” was agreed for medium risk back, knee and multisite pain, but not for neck or shoulder pain. “Atypical analgesia (e.g. Amitriptyline, Pregabalin, Gabapentin)” was agreed for medium risk back, multisite and neck pain, but not for knee or shoulder.

#### High risk

Nine treatment options reached consensus for back pain, ten for knee pain, nine for multisite pain, nine for neck pain, and eight for shoulder pain. Six treatment options were agreed across all five pain presentations for patients at high risk: “consider referral to physiotherapy (all modalities)”; “consider referral to MSK interface clinic”; “consider referral to psychosocial intervention or multidisciplinary pain management service”; “address comorbidities, distress and frailty”; “opioids”; and “consider referral to expert patient programme”. “Occupational Health/work place assessment and advice” was agreed for patients at high risk with back, knee and neck pain, respectively, but not for shoulder or multisite pain. “Atypical analgesia” reached consensus for all presentations with the exception of knee. “Corticosteroid injection” reached consensus only for knee and shoulder pain for high risk patients. “Refer for surgical opinion” was included for all presentations for high risk except for multisite pain; however, multisite pain was the only presentation for which “refer to rheumatology” was included for high risk. Two other treatment options were agreed only for one of the pain presentations and none of the others: “personalised exercise programmes” (multisite) and “refer for lifestyle intervention e.g. dietician, slimming world etc.” (knee). The full list of agreed treatment options for all five pain presentations across the three risk subgroups is displayed in Table 4. The results are summarised in Table 5, below:

**Table 5:** Summary of results: Discursive summary of the agreed treatment options across all five pain presentations and three risk subgroups: low, medium and high

Low risk	Self-management education/ advice; simple pain medications limited to those available over the counter.
Medium risk	In addition to options at low risk, may consider options such as: onward referral, e.g. physiotherapy, MSK interface clinic; or prescribing atypical analgesia.
High risk	In addition to options at low and medium risk, may consider options such as: referral to psychosocial intervention or pain management service; refer to expert patient programme; refer for surgical opinion; address comorbidities, distress and frailty.

## Discussion

This study generated an acceptable level of consensus (which we defined as an average across all participants of > 3.5 out of 7 on a likert scale (i.e. > 50%) for each treatment option, as discussed further below), among a group of practitioners who work with MSK pain patients about appropriate matched treatment options for each of the five most common MSK pain presentations (back, knee, shoulder, neck and multisite pain) according to the patient’s risk of persistent disabling pain. Seventeen treatment options in total reached our predefined level of consensus – four for patients at low risk, 10 for medium risk and 15 for high risk, with some variation observed in treatment options reaching consensus across different MSK pain presentations. It was reassuring to note that as the risk of persistent disabling pain increased, the suggested treatment options also increased in both number and intensity. This would be expected given that patients at higher risk will likely have more complex needs− either due to high pain intensity, psychological or social factors, or a combination of these− and it is therefore appropriate for GPs to have a range of treatment options to choose from depending on the individual needs of the patient, including more intensive or invasive options if necessary. The final agreed matched treatment options appear to be both clinically appropriate and in keeping with the risk profiles for each of the three risk subgroups.

### Comparison across MSK pain presentation and risk subgroup

The agreed treatment options for patients at low risk principally involve GP-supported self-management, and do not include referral for further treatment, reflecting the aim of stratified care to avoid over-treating patients with a good prognosis [24]. The additional treatment option agreed only for low risk multisite pain – “refer to supported self-management and locally available community resources e.g. walking group, exercise on prescription” – may reflect the additional complexity of managing pain at more than one body region site, with the participants believing that these patients would benefit from further support in addition to that provided by the GP.

As well as the treatment options agreed for low risk, medium risk included the addition of onward referral options across all five MSK pain sites: “consider referral to physiotherapy” and “consider referral to MSK interface clinic”. Including options for onward referral reflects the increased need of patients at medium risk, who may experience higher levels of pain and greater functional limitations compared to low risk patients. There was also some variation in agreed treatment options for patients with different MSK pain-site presentations who are at medium risk of poor outcome. For instance, “consider referral to psychosocial intervention or pain management service” was included for back and multisite pain, but not for the other three pain sites; this may reflect the higher prevalence of psycho-social issues associated with these pain sites, e.g. both are strongly associated with an increased risk of developing co-morbid depression [25, 26]. Other agreed treatment options that differed across pain sites included “atypical analgesia”, which reached consensus for patients at medium risk with back, multisite and neck pain, but not for those with knee or shoulder pain; which is likely to reflect the greater prevalence of radicular pain from these three sites. A number of guidelines suggest that atypical analgesia is more effective for neuropathic pain and following discussion amongst the participants this was further simplified to being recommended for patients with radicular pain. Two treatment options were agreed only in relation to one of the five pain sites at medium risk: “corticosteroid injection” (shoulder), and “refer to rheumatology” (multisite). Again these results appear clinically appropriate: administering an injection is a common first-line treatment for shoulder pain; and the option to refer to rheumatology for multisite pain patients takes into account the importance of identifying in primary care early indications of inflammatory arthritis, in order to reduce diagnostic delay [27].

There was some overlap in the matched treatment options that reached consensus for patients at medium and high risk of poor outcome, although additional options were agreed for patients at high risk across all five pain presentations. Two treatment options reached consensus for all five MSK pain presentations for patients at high risk, but did not reach consensus for any of the MSK pain presentations for patients at medium risk: “opioids” and “refer to expert patient programme”. That opioids were agreed only for patients at high risk of poor outcome reflects current UK practice guidelines– primary care clinicians are encouraged to prescribe non-opioid based analgesia where possible, and only prescribe opioids for patients with high pain levels or who have an inadequate response to paracetamol and/or nonsteroidal anti-inflammatory drugs [14]. The expert patient programme places focus on self-management of long-term conditions and therefore may be appropriate for some high risk patients given their poorer prognosis, and subsequent higher probability of experiencing long-term disability. “Refer for surgical opinion” reached consensus only for patients at high risk, for all MSK pain sites except multisite pain; whilst “refer to rheumatology” was agreed for patients with multisite pain only. This again reflects the different perspectives from this group of practitioners about how best to manage multisite pain – and particularly widespread pain – in comparison to single site pain. “Corticosteroid injection” reached consensus for patients with knee and shoulder pain at high risk, but not for patients with MSK pain at the other three sites; again reflecting common practice of injecting peripheral joints [28]. “Refer for lifestyle intervention e.g. dietician, slimming world etc.” reached consensus only for patients at high risk with knee pain and not for any of the other four pain presentations. This reflects the evidence base regarding the association between obesity and knee joint pain [29] and the functional difficulties resulting from knee pain that can hinder patients’ efforts to engage in physical activity.

### Strengths and limitations

This study innovatively combined NGT with pre-elicitation techniques of patient vignettes derived from epidemiological data and supported by a rigorous review of the available best evidence [18] (see Fig. 2). The multidisciplinary nature of the sample of participants was also an important strength as this allowed access to a varied range of expertise in the field of MSK pain. It was felt that this would lead to less restricted results than might have been found had the participants been made up of GPs alone. Whilst this represents a strength of the study, including a wide range of clinicians made it necessary to use a lower threshold to determine consensus than might have been the case if the group comprised clinicians from a single discipline, who may have more homogenous views towards treatment [22]. The > 50% cut-off point used for signalling agreement may be viewed as a limitation of the study. However, our aim was not to identify the single ‘best’ treatments that would be appropriate for all patients in a given risk subgroup, but to identify a suite of treatment options that can be recommended to GPs, so that when these options are used in consultations as part of the new stratified care model, this can allow for professional and patient shared decision-making. Patients stratified into the same subgroup may have different treatment needs despite having similar risk profiles, and certain matched treatment options may be more or less appropriate for an individual patient; therefore, a high threshold for signalling agreement would not have been appropriate. Using a higher threshold, e.g. > 75%, would have resulted in fewer recommended treatment options. Taking high risk knee pain as an example using a > 75% cut off, in addition to the two generic treatments agreed across all three risk subgroups, only two further treatment options would have gained consensus: “consider referral to physiotherapy”, and “consider referral to MSK interface clinic”. The other eight treatment options would have been excluded; similar findings regarding the restrictive nature of using a higher consensus threshold are reported in previous NGT studies [30, 31]. The decision to use a > 50% cut-off was further reinforced through agreement from the study participants as to the suitability of the final treatment options which were sent to them via email following the consensus group meetings (see Methods, above).

The UK focus of the study represents both a strength and weakness. Whilst the geographical spread of participants enabled us to produce a list of recommended treatment options that is likely to have good representativeness across the UK, the applicability beyond the UK depends on treatment availability in other settings. However, the majority of treatment options that reached consensus are not UK-specific; therefore, findings can have wider applicability beyond the UK context and may be useful for informing research and practice in other settings.

A limitation is that the use of a consensus group methodology resulted in the exclusion of newer or less widely used treatment options. However, as previously mentioned, we included a wide range of clinicians to avoid missing any treatment options considered to be best practice, even if not always supported by available evidence. It was not considered to be appropriate to recommend untested treatment options; these treatment options would require further research and testing. A further limitation of this study is that we did not include patient representatives in our expert groups. Whilst we recognise that expert patients would have valuable input for decisions about treatment options, it was felt that this study was specifically about clinical expertise and therefore was limited to clinician input. Extensive patient representation has been included in the broader research programme [6], and the development and testing of the stratified primary care intervention.

## Conclusion

A group of multi-disciplinary practitioners generated consensus about appropriate matched treatment options for risk subgroups of primary care MSK pain patients (at low, medium and high risk of persistent disabling pain). This information informed the matched treatment options in a trial of stratified primary care for patients with MSK pain. The results can also be helpful for clinicians and patients to support greater consistency in the management of MSK pain, addressing the issue highlighted earlier that current variability in management can lead to some patients, at least initially, receiving suboptimal care including inappropriate treatment referrals. Supporting greater consistency in management can help ensure the right patient receives the right treatment at the right time.

## Footnotes

1 MSK Interface Clinics are a multi-professional service for patients with non-inflammatory and non-surgical MSK disease, at the interface between primary and secondary care. Clinicians aim to assess and diagnose MSK problems and agree a management plan in conjunction with the patient. Patients can be referred to interface clinics by general practitioners and physiotherapists.

## References

[1] Vos T, Allen C, Arora M, Amare AT, Lalloo R (2016). Global, regional, and national incidence, prevalence, and years lived with disability for 310 diseases and injuries, 1990–2015: a systematic analysis for the global burden of disease study 2015. Lancet.

[2] Jordan K, Kadam U, Hayward R, Porcheret M, Young C, Croft P (2010). Annual consultation prevalence of regional musculoskeletal problems in primary care: an observational study. BMC Musculoskelet Disord.

[3] Maserejian NN, Fischer MA, Trachtenberg FL, Yu J, Marceau LD, McKinlay JB, Katz JN (2014). Variations among primary care physicians in exercise advice, imaging, and analgesics for musculoskeletal pain: results from a factorial experiment 2014. Arthritis Care Res.

[4] Darlow B, Dean S, Perry M, Mathiesond F, Baxter GD, Dowell A (2014). Acute low back pain management in general practice: uncertainty and conflicting certainties. Fam Pract.

[5] Alami S, Boutron I, Desjeux D, Hirschhorn M, Meric G, Rannou F, Poiraudeau S (2011). Patients’ and practitioners’ views of knee osteoarthritis and its management: a qualitative interview study. PLoS One.

[6] Saunders B, Bartlam B, Foster NE, Hill JC, Cooper V, Protheroe J (2016). General practitioners’ and patients’ perceptions towards stratified care: a theory informed investigation. BMC Fam Pract.

[7] Buchbinder R, Staples MP, Shanahan EM, Roos JF (2013). General practitioner Management of Shoulder Pain in comparison with rheumatologist expectation of care and best evidence: an Australian National Survey. PLoS One.

[8] Cottrell E, Roddy E, Rathod T, Porcheret M, Foster NE (2016). What influences general practitioners’ use of exercise for patients with chronic knee pain? Results from a national survey. BMC Fam Pract.

[9] Foster NE, Hill JC, O’Sullivan P, Hancock M (2013). Stratified models of care. Best Pract Res Clin Rheumatol.

[10] Hill JC, Whitehurst DG, Lewis M, Bryan S, Dunn KM, Foster NE, Konstantinou K, Main CJ, Mason E, Somerville S, Sowden G, Vohora K, Hay EM (2011). Comparison of stratified primary care management for low Back pain with current best practice (STarT Back): a randomised controlled trial. Lancet.

[11] Foster NE, Mullis R, Hill JC, Lewis M, Whitehurst DG, Doyle C, Konstantinou K, Main CJ, Somerville S, Sowden G, Wathall S, Young J, Hay EM (2014). Effect of stratified care for low Back pain in family practice (IMPaCT Back): a prospective population-based sequential comparison. Ann Fam Med.

[12] Whitehurst DG, Bryan S, Lewis M, Hay EM, Mullis R, Foster NE (2015). Implementing stratified primary care management for low back pain: cost-utility analysis alongside a prospective, population-based, sequential comparison study. Spine.

[13] Hill JC, Dunn KM, Lewis M, Mullis R, Main CJ, Foster NE, Hay EM (2008). A primary care back pain screening tool: identifying patient subgroups for initial treatment. Arthritis Care Res.

[14] NICE (National Institute for Health and Care Excellence) Guidance. https://www.nice.org.uk/guidance/conditions-and-diseases/musculoskeletal-conditions. Accessed 14 Apr 2018.

[15] Green DJ, Lewis M, Mansell G, Artus M, Dziedzic KS, Hay EM, Foster NE, van der Windt DA (2018). Clinical course and prognostic factors across different musculoskeletal pain sites: a secondary analysis of individual patient data from randomised clinical trials. Eur J Pain.

[16] Campbell P, Hill JC, Protheroe J, Afolabi E, Lewis M (2016). Keele aches and pains study protocol: validity, acceptability and feasibility of the Keele STarT MSK tool for subgrouping musculoskeletal patients in primary care. J Pain Res.

[17] Jones J, Hunter G (1995). Consensus methods for medical and health services research. BMJ.

[18] Babatunde OO, Jordan JL, Van der Windt DA, Hill JC, Foster NE, Protheroe J (2017). Effective treatment options for musculoskeletal pain in primary care: a systematic overview of current evidence. PLoS One.

[19] Delbecq AL, van de Ven AH, Gustafson DH (1975). Group techniques for problem planning.

[20] Gonzales CK, Leroy G (2011). Eliciting user requirements using appreciative inquiry. Empir Softw Eng.

[21] Leape LL, Hilborne LH, Kahan JP, Stason WB, Park RE, Kamberg CJ (1992). Coronary artery bypass graft: a literature review and ratings of appropriateness and necessity.

[22] Coulter I, Adams A, Shekelle P (1995). Impact of varying panel membership on ratings of appropriateness in consensus panels: a comparison of a multi- and single disciplinary panel. HSR.

[23] Murphy MK, Sanderson CBD, Black NA, Askham J, Lamping DL, Marteau T, McKee CM (1998). Consensus development methods, and their use in clinical guideline development. Health Technol Assess.

[24] Hingorani AD, van der Windt DA, Riley RD, Abrams K, Moons KGM (2013). Prognosis research strategy (PROGRESS) 4: stratified medicine research. BMJ.

[25] Bener A, Verjee M, Dafeeah EE, Falah O, Al-Juhaishi T, Schlogl J, Sedeeq A, Khan S (2013). Psychological factors: anxiety, depression, and somatization symptoms in low back pain patients. J Pain Res.

[26] Crofford LJ (2015). Psychological aspects of chronic musculoskeletal pain. Best Pract Res Clin Rheumatol.

[27] Villeneuve E, Nam JL, Bell MJ, Deighton CM, Felson DT (2013). A systematic literature review of strategies promoting early referral and reducing delays in the diagnosis and management of inflammatory arthritis. Ann Rheum Dis.

[28] Artus M, van der Windt DA, Afolabi EK, Buchbinder R, Chesterton LS, Hall A, Roddy E, Foster NE (2017). Management of shoulder pain by UK general practitioners (GPs): a national survey. BMJ Open.

[29] Okifuji A, Hare BD (2015). The association between chronic pain and obesity. J Pain Res.

[30] Naylor CD, Basinski A, Baigrie RS, Goldman BS, Lomas J (1990). Placing patients in the queue for coronary revascularization: evidence for practice variations from an expert panel process. Am J Publ Health.

[31] Rubin G, De Wit N, Meineche-Schmidt V, Seifert B, Hall N, Hungin P (2006). The diagnosis of IBS in primary care: consensus development using nominal group technique. Fam Pract.

